# Evaluation of the applicability of GARDskin to predict skin sensitizers in extracts from medical device materials

**DOI:** 10.3389/ftox.2024.1320367

**Published:** 2024-03-12

**Authors:** Rose-Marie Jenvert, Olivia Larne, Angelica Johansson, Mattias Berglin, Emma Pedersen, Henrik Johansson

**Affiliations:** ^1^ SenzaGen AB, Lund, Sweden; ^2^ RISE Research Institutes of Sweden AB, Borås, Sweden

**Keywords:** gard, skin sensitization, *in vitro*, NAMs, medical device, ISO 10993, biocompatibility

## Abstract

Biocompatibility testing of medical devices is governed by the ISO 10993 series of standards and includes evaluation of skin sensitization potential of the final product. A majority of all medical devices are tested using *in vivo* methods, largely due to the lack of *in vitro* methods validated within the applicability domain of solid materials. The GARDskin method for assessment of chemical skin sensitizers is a validated method included in the OECD Test Guideline 442E, based on evaluation of transcriptional patterns of an endpoint-specific genomic biomarker signature in a dendritic cell-like cell, following test chemical exposure. The current study aimed to evaluate the applicability of GARDskin for the purpose of testing solid materials by incorporation of extraction procedures described in ISO 10993-12:2021, as well as to demonstrate the functionality of the proposed protocols, by testing of custom-made materials spiked with sensitizing agents. It was shown that GARDskin is compatible with both polar and non-polar extraction vehicles frequently used for the purpose of medical device biological testing. Further, exploring three different material types spiked with up to four different sensitizing agents, as well as three unspiked control materials and commercial reference products, it was shown that the method correctly classified all evaluated test materials. Taken together, the data presented suggest that GARDskin may constitute a valid alternative to *in vivo* experimentation for the purpose of skin sensitization assessment of medical devices.

## Introduction

Medical device toxicology is currently being redefined, transitioning from a process that largely relies on the results of animal testing, to one which is increasingly focused on the use of *in vitro* and *in chemico* methods for evaluation of the biological safety of devices (Kerecman Myers et al., 2017). The biological evaluation of medical devices is governed by the ISO 10993 series of standards, where the framework is described in ISO 10993-1: 2018. (ISO 10993-1, 2018). The biological endpoints (i.e., adverse effects) related to skin exposure that have to be evaluated for all medical devices in direct or indirect contact with the body include skin irritation (ISO 10993-23, 2021) and skin sensitization (ISO 10993-10, 2021). An *in vitro* test method to assess skin irritation of medical devices has been validated and included in ISO 10993-23 in 2021 and is now the preferred choice for irritation testing and is increasingly accepted on many markets worldwide. (De Jong et al., 2018). However, an *in vitro* test method to assess skin sensitization potential of medical devices or materials has not been sufficiently qualified. Rather, such testing is conventionally performed *in vivo*, primarily using the Guinea Pig Maximization Test (GPMT) (Magnusson and Kligman, 1969) or the Buehler Occluded Patch Test (Buehler, 1965).

The immunobiological mechanisms leading to skin sensitization are today well understood and summarized as an Adverse Outcome Pathway (AOP) (OECD, 2014). To date, several mechanistically based new approach methodologies (NAM) for *in vitro* assessment of chemical skin sensitizers have been validated and regulatory accepted for use within certain applicability domains. These methods address the first three of the four key events (KE) of the AOP and are described in OECD Test Guidelines (TG) 442C, 442D and 442E, respectively (OECD, 2022b; OECD, 2023a; OECD, 2023b). There is now an interest in qualifying these methods for the assessment of skin sensitization of medical devices, where the framework for such qualification has been outlined in a recent guidance document (ISO/TS 11796, 2023).

Several next-generation NAM:s are emerging, with the potential of both broadening and sharpening the applicability of *in vitro* methods. One such method is the Genomic Allergen Rapid Detection (GARD) assay for assessment of chemical skin sensitizers (GARDskin) (OECD, 2023b), which was regulatory accepted and included in the OECD TG 442E in 2022. The GARDskin method evaluates the transcriptional patterns of an endpoint-specific genomic biomarker signature (GPS) (Johansson et al., 2011; OECD, 2022a) in a dendritic cell-like cell line exposed to test chemicals, which allows for machine-learning assisted classifications based on high-dimensional gene expression data, monitoring a wide range of immunobiological mechanisms associated with the AOP for skin sensitization.

Of importance, all available OECD *in vitro* methods for skin sensitization were developed and validated for assessment of neat chemicals. For these methods to be qualified for assessment of complex and dilute mixtures, metals, and solid materials, additional developments and validating exercises are required. In the case of the biological evaluation of medical devices, all testing of solid materials is to be performed on extracts containing a mixture of chemicals extracted from the material, by the use of both a polar and non-polar solvent (i.e., extraction vehicles), in order to capture clinically relevant leachables (ISO 10993-12, 2021). Thus, any *in vitro* test system utilized for such purposes must be both compatible with such solvents, at sufficient concentrations, and be sensitive enough in order to detect the presence of skin sensitizing agents in a complex and dilute formulation.

Here, the progress in adapting the conventional GARDskin method to comply with requirements imposed by the ISO 10993 series of standards is presented. The assay’s compatibility with polar and non-polar solvents has been evaluated. Presented data also demonstrate the functionality of ISO 10993-12:2021 protocols for extraction of materials frequently used in medical devices, spiked with weak, moderate and strong sensitizers and the ability of the cellular system to accurately detect the presence of extracted chemicals from the materials.

## Materials and methods

### Test materials and controls

The spiked test materials was produced by the Research Institutes of Sweden (RISE). Four different sensitizing agents were used as spikes during production: 2-Aminophenol, Propyl gallate, Cinnamic aldehyde and Phenyl benzoate. All chemicals were purchased from Sigma-Aldrich (St. Louis, MO, US). Additional details of each sensitizing agent are provided in Table 1. The rationale for selection of sensitizing agents was based on the following selection criteria. Firstly, the chemicals should represent a range of sensitizing potencies, including weak, moderate and strong sensitizers. Secondly, the chemicals should have available reference data from historical assessment using the conventional GARDskin method. Lastly, the chemicals should, if possible, have been used in similar work (Coleman et al., 2015; Pellevoisin et al., 2021) in order to facilitate direct comparisons of results. The sensitizing agents were introduced in silicone (MED-2000, NuSil Technology, Carpinteria, CA) and thermoplastic polyurethane (TPU, Coim Laripur^®^ LPR 7560 EG, Telko, Sweden) at a final concentration of 10%, similar to the materials described and used in similar work (Coleman et al., 2015; 2018). In addition, commercially available materials, approved for the medical device market, were included in the study as negative reference materials. For this purpose, a silicone tube (EC60001), a TPU tube (Estane 58,277) and a polyvinyl chloride (PVC) tube (RB3 NDG), were all acquired from Medizintechnik Promedt GmbH (Tornesch, Germany). The vehicles used for extraction were Super Refined Olive Oil-LQ-(MH) (CAS 8001-25-0) (Croda, East Yorkshire, UK), Sesame oil (CAS 8008-74-0) (Sigma Aldrich, St, Louis, MO) and 0.9% saline (G-Biosciences, St. Louis, MO) supplemented with 1% Penicillin-Streptomycin Solution (PEST) (100x stock solution, 10,000 units/mL Penicillin, 10,000 μg/mL Streptomycin) (Biowest, Riverside, MO). The positive control, p-Phenylenediamine (PPD) (CAS No. 106-50-3) (Sigma Aldrich, St. Louis, MO) was freshly prepared as 1,000x stock solutions in DMSO and further diluted in respective extraction vehicles prior to in-well cellular exposures, as further described below.

**TABLE 1:** Details of sensitizing agents used for production of custom materials^a^.

Chemical ID	CAS	MW	LogP	MoA^b^	Pre-hapten	LLNA^c^	Human^c^
2-Aminophenol	95-55-6	109.13	0.62	MA	Yes	1 (1A)	*NA*
Propyl gallate	121-79-9	212.2	1.8	MA	Yes	1 (1A)	*NA*
Cinnamic aldehyde	14,371-10-9	132.16	1.9	MA	No	1 (1A)	1 (1A)
Phenyl Benzoate	93-99-2	198.22	3.59	AT	No	1	1 (1B)

a Values are collected from Annex 2 of the Supporting Document to the OECD, Guideline 497 on Defined Approaches for Skin Sensitisation (OECD, 2021), unless indicated otherwise.

b MoA: mechanism of action, MA: michael acceptor, AT: Acyl Transfer. Values are collected from Roberts et al., 2007.

c Reference values of sensitizing properties, according to the United Nations Globally Harmonized System of Classification and Labelling of Chemicals (UN GHS) (United Nations, 2015).

### Production of silicone-based test materials

To facilitate dispersion in silicone films the sensitizing agents were first dissolved in tetrahydrofuran (THF) (CAS No. 109-99-9) (Sigma-Aldrich) at 1 g/mL. Of each solution with sensitizing agent 500 µL was added to 4.7 g of Silicone, MED-2000 (USP class VI Medical Grade), to obtain a final pre-cured concentration of sensitizer of 9.6% (w/w). The reference silicone, used as negative control material, was impregnated with 500 µL THF without any sensitizing agent added. Uncured silicone samples were mixed using a Speed-Mixer DAC150 FV at 3,500 rpm for 2 min. Silicone mixtures were dispensed into a metal mold (110 × 110 × 2 mm) clamped between two Teflon sheets under pressure and cured at ambient temperature and humidity under pressure (1 kPa) for 72 h. During curing, acetic acid is released resulting in a final concentration of sensitizing agent in the silicone film of 10% (w/w). Due to the volatility of THF, the vehicle is not expected to remain in the silicone film after curing. Following curation, high quality materials and good dispersions of sensitizing agents were ensured by visual inspection and characterized with Attenuated Total Reflection Fourier Transform Infrared Spectroscopy (ATR-FTIR), using a Nicolet 6700 FT-IR Spectrometer (Thermo Scientific, Waltham, MA, US) (data not shown). All test materials were stored at 4°C.

### Production of TPU-based test materials

TPU pellets (USP Class VI Medical Grade) were cryomilled in a Retsch ZM200 Ultra Centrifugal Mill (Retsch, Dusseldorf, Germany) to obtain a TPU powder. The TPU powder was thoroughly mixed with skin sensitizing agents at 10% (w/w) using a spatula. The reference TPU film, used as negative control material, was prepared using identical TPU without any impregnating additions. The prepared mixtures were dispensed into a metal mold (110 × 110 × 2 mm) clamped between two Teflon sheets under pressure, using a Fontijne LabEcon 300 hydraulic laboratory press (Fontinje, Rotterdam, Netherlands) and was cured in the press at 180°C, pre-heated for 2 min at the almost closed position before pressing at 50 kN for 1 min and then cooled to 45°C at maintained press force. Following curation, high quality materials were confirmed by visual inspection and ATR-FTIR, as described above (data not shown). All test materials were stored at 4°C.

### Test material extractions

The test material was prepared according to ISO 10993-12 (ISO 10993-12, 2021). In short, 0.2 g of the material was incubated, with rotation, at 37°C (±1°C) for 72 h (±2 h) in 1 mL of 0.9% saline supplemented with 1% PEST, 1 mL Super Refined Olive Oil-LQ-(MH) or 1 mL Sesame oil in borosilicate glass vials with caps of Teflon (C405-1) (Thermo Scientific, Waltham, MA). The test material was extracted in three replicates in each vehicle. After the extraction, the test material extracts were used for cell exposure experiments the same day. Vehicle controls (saline, olive oil and sesame oil) were incubated at identical conditions as the test material and were included in each GARD experiment.

### GARDskin medical device protocol

The protocols of the conventional GARDskin assay have been previously published (EURL ECVAM, 2021) and summarized in OECD test guideline (OECD, 2023b). In short, the GARDskin utilizes the human myeloid leukemia SenzaCell cell line (ATCC Depository PTA-123875) as an *in vitro* model for dendritic cells (DC), thus monitoring the immunological activation of DC in response to xenobiotics following test item exposure.

Initial exposure experiments are performed in a titrated range of test item concentrations in order to evaluate its cytotoxic properties. Based on the dose-response relationship of exposure concentrations and cell viability, test item-specific GARD input concentrations are established at low-to non-toxic levels, which is used for downstream testing. Specifically, any test item exhibiting cytotoxic properties are assayed at the concentration yielding ∼90% relative cell viability, referred to as the Rv90 concentration. Correspondingly, non-cytotoxic test items are assayed at the top concentration of the explored titration range, which for neat chemicals is set at a default value of 500 µM.

Following repeated exposure experiments at the GARD input concentration, RNA is isolated from exposed cell cultures in three independent experiments. Here, independent experiments refer to experiments separated in time, with freshly and separately prepared cell cultures, samples and reagents, thus giving rise to biological replicate samples. The gene expression profile, i.e., mRNA levels of the genes in the GARDskin GPS (Johansson et al., 2011), is quantified using NanoString nCounter technology (Geiss et al., 2008) and the skin sensitizing hazard property of the test item is predicted using the GARDskin prediction model, based on a Support Vector Machine (SVM) (Cortes and Vapnik, 1995) prediction algorithm, appropriately trained and frozen during assay development (Forreryd et al., 2016). The prediction algorithm assigns individual samples with decision values (DV), the signs of which are evaluated for hazard classification; any test item with a positive mean DV is classified as a skin sensitizer. Specific details of the complete analysis pipeline, including a background on the identification and immunological relevance of genomic biomarkers of the GPS, data handling, normalization procedures and an in-depth description of the prediction algorithm, are publicly available elsewhere (OECD, 2022a).

The term test item is used here to describe the entity of investigation in any GARDskin assay. However, the GARDskin assay was designed for, and validated for, assessment of neat monoconstituent chemical compounds. The following protocol adaptations were introduced in order to successfully assess sensitizing hazard of chemicals extracted from solid materials, while also complying with relevant ISO 10993 standards.

Firstly, the assay compatibility of a selected set of extraction vehicles, as described in sections above, was evaluated. To prepare for assessment of dilute extracted chemicals in complex formulations, extraction vehicle exposure concentrations were sought to be maximized, while maintaining steady state cell cultures in terms of cell viability and a non-detectable impact on GARDskin classifications. For exposures of oils to cell culture-wells, oil were added to the top of the cell medium, without additional attempts to mix the oil-water phases.

Secondly, when evaluating cytotoxic properties of test material extracts, the top concentration in the titration range was set to correspond to the optimized concentration of the respective extraction vehicles. Subsequent establishment of test material extract-specific GARD input concentrations was done by application of identical decision criteria as used within the conventional GARDskin assay. Of note, however, when testing test material extracts, as opposed to neat chemicals, deviating input concentrations are accepted in downstream repeated exposures giving rise to triplicate RNA samples. This protocol design was introduced to take into account two additional sources of variability; Firstly, test materials cannot be assumed to be 100% homogenous. Depending on the specific parts of the test materials being prepared for extraction, different compositions of test material extracts may be achieved. Secondly, as also considered and described in ISO 10993-12:2021, extraction procedures cannot be assumed to be 100% reproducible, as varying degrees of extraction efficiency may be expected.

Thirdly, the positive control of the conventional GARDskin assay (PPD) was administered in the same extraction vehicles used for administration of test items, at identical exposure conditions and vehicle concentrations. Similarly, the blank extraction vehicles, at identical exposure conditions, were considered negative controls.

Lastly, the prediction model was adapted to mirror the dual testing of polar and non-polar solvents. A test material with a positive mean DV from either, or both, of the polar and non-polar test material extracts is classified as a skin sensitizer.

Thus, the conventional GARDskin assay protocols with the adaptations listed above forms the protocols henceforth referred to as the GARDskin Medical Device protocols, which were used to generate all data in this study. A list of key experimental steps and technical components comparing the conventional GARDskin method with the GARDskin Medical Device protocols is presented in Table 2.

**TABLE 2:** Comparison of key experimental steps and technical components of the OECD TG442E GARDskin method and the GARDskin Medical Device protocols.

Experimental step/technical component	TG 442E GARDskin	GARDskin medical device
Cellular test system	SenzaCell cell line (ATCC Depository PTA-123875)	SenzaCell cell line (ATCC Depository PTA-123875)
Test item^a^	Pure monoconstituent chemical	Test material extract
Vehicles/extraction vehicles^a^	DMSO, dH_2_O (Final in-well concentration 0.1%)	Olive oil, Sesame oil, saline (Final in-well concentration 10%)
Default top concentration for cytotoxicity screening^1^	500 μM (pure test item concentration)	10% (test material extract concentration)
GARDskin input concentration	Rv90-concentration or default top concentration. Identical in all replicate samples	Rv90-concentration or default top concentration. Allowed to vary across replicate samples
Positive control	PPD, administered in accepted vehicle	PPD, administered in accepted vehicle
Negative control	Blank vehicle at in-well concentration	Blank vehicle at in-well concentration
Endpoint measurement	Quantified gene expression of the GARDskin GPS	Quantified gene expression of the GARDskin GPS
Analysis pipeline	Default GARDskin analysis pipeline^b^	Default GARDskin analysis pipeline^b^
Prediction model^1^	Mean DV ≥ 0: sensitizer	Mean DV in any one vehicle ≥0: sensitizer

^a^ Notable difference.

^b^ For details, cf OECD, 2022a.

## Results

### Compatibility of extraction vehicles with the test system

**FIGURE 1:**
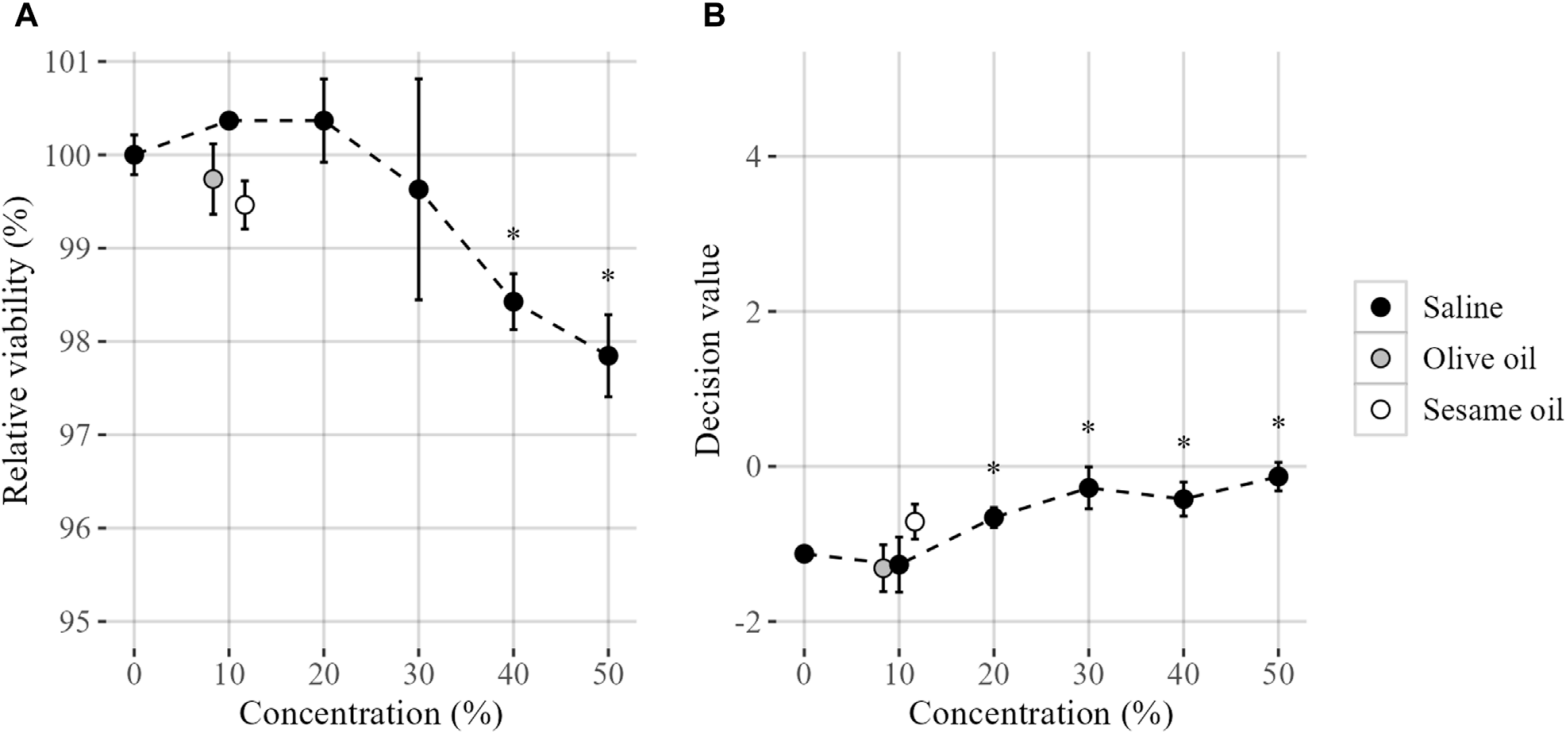
Compatibility of extraction vehicles on steady state cell cultures, evaluated by **(A)** the relative viability and **(B)** GARDskin Decision Values. Mean values (n = 3) are presented by plotted datapoints, and standard deviation are represented by error bars. Statistical significance (Student’s t-test, *p* < 0.05) of deviating values compared with unstimulated control are indicated by an asterisk (*). The figures are produced with a jitter function for increased visibility. Tested concentrations were 0, 10, 20, 30, 40% and 50% (saline) and 0% and 10% (olive and sesame oil).

The successful applicability of GARDskin to assess skin sensitizing potential of extracted chemicals from solid materials is dependent on the test system’s compatibility with both polar and non-polar vehicles used for extraction. To this end, the compatibility of saline, olive oil and sesame oil with the test system was evaluated, with results summarized in Figure 1.

Firstly, a range of in-well saline concentrations were evaluated. The saline was well tolerated by the SenzaCell cell line, with a non-detectable impact of relative cell viability, as compared to unstimulated cells, using up to 30% saline in-well. However, further increase of the saline concentration results in a small, but statistically significant, decrease in relative cell viability.

The impact of saline on the GARDskin prediction algorithm output, i.e., the DV:s generated by the SVM classifier, was also investigated. While negative classifications are generated throughout the titration range, a statistically significant impact is detectable on saline concentrations of 20% and above, compared to unstimulated cells. Thus, it was concluded that a top concentration of 10% saline is compatible with the test system, with maintained steady-state cell cultures.

The compatibility with the non-polar vehicles olive oil and sesame oil was then evaluated, guided by the results of the saline evaluation. It was confirmed that both olive and sesame oil were tolerated by the test system at the proposed concentration (10%), again by monitoring the impact of relative cell viability compared to unstimulated cells, as well as the potential impact on baseline GARDskin DV:s.

Following these findings, the GARD skin Medical Device protocols adopted a procedure in which all test item exposures are administrated with an in-well extraction vehicle concentration of 10%. Any dilutions of test material extracts and controls required in order to mitigate cytotoxicity are done using the corresponding blank vehicle, so that all cell exposures, irrespective of dilution factor of the test item, are performed with an identical and non-detectable background signal from the extraction vehicle.

### GARDskin predictions of test materials and controls

In the absence of readily available medical device-associated materials with sensitizing properties, the functionality of the extraction procedures and the ability of the GARDskin prediction model to accurately detect the presence of sensitizing agents in obtained extracts was demonstrated using customized test materials. First, a silicone material (MED-2000) was spiked with 2-aminophenol, propyl gallate, cinnamic aldehyde and phenyl benzoate and assayed along with an unspiked negative control material. Similarly, a TPU material was spiked with 2-aminophenol and cinnamic aldehyde and assayed along with an unspiked negative control material. Lastly, these customized test materials were complemented with commercially available counterparts, i.e., tubes made out of silicone and TPU, as well as a PVC material. All eleven test materials were subjected to extraction procedures in adherence with ISO standards, using saline and olive oil as polar and non-polar vehicles, respectively. In addition, all but the customized TPU materials were extracted using sesame oil as a complimentary polar vehicle.

All resulting test items (i.e., extracts) were assayed according to the GARDskin Medical Device protocols, as established above. In an initial step, the cytotoxic properties of each test item were evaluated. As defined, all non-cytotoxic test items were assayed at the default top concentration of 10%. Any test item inducing cytotoxicity was diluted in the respective extraction vehicle, to the concentration inducing 90% relative cell viability (defined as the Rv90 concentration), before being administered to in-well cell exposures. All final test item concentrations used for downstream analysis are summarized in Table 3.

**TABLE 3:** GARD input concentration (%) of test items in each respective extraction vehicle.

Test material^a^	Concentration (%)^b^		
	Saline	Olive oil	Sesame oil
MED-2000	10, 10, 10	10, 10, 10	10, 10, 10
MED-2000 + 2AP	0.1, 0.1, 0.1	1.25, 1, 1	1, 1, 1
MED-2000 + PG	1, 1.25, 1	1.25, 1.5, 1.5	1.25, 1.25, 1
MED-2000 + CA	3.5, 2.67, 3.5	3.5, 3.75, 3.75	4, 3.75, 4
MED-2000 + PB	10, 10, 10	10, 10, 10	10, 10, 10
TPU	10, 10, 10	10, 10, 10	NA
TPU + 2AP	1, 1, 1	0.1, 0.1, 0.1	NA
TPU + CA	1, 1.25, 1.25	0.25, 0.25, 0.25	NA
Silicone tube	10, 10, 10	10, 10, 10	10, 10, 10
TPU tube	10, 10, 10	10, 10, 10	10, 10, 10
PVC tube	10, 10, 10	10, 10, 10	10, 10, 10
Negative control	10, 10, 10	10, 10, 10	10, 10, 10
Positive control^c^	75, 75, 75 (µM)	90, 90, 90 (µM)	100, 100, 100 (µM)

^a^ 2AP: 2-aminophenol, PG: propyl gallate, CA: cinnamic aldehyde, PB: phenyl benzoate.

^b^ Note that all cell exposures were performed in identical conditions, i.e., with an identical in-well vehicle concentration of 10%. Herein presented concentrations represent the actual test item concentrations, following dilutions (in each respective vehicle) in order to mitigate cytotoxic effects.

^c^ The positive control (PPD) has a known molarity, which has been considered when presenting data. All dilutions were made in respective vehicles, and cell exposures were performed in identical conditions (10% in-well vehicle concentration).

**FIGURE 2:**
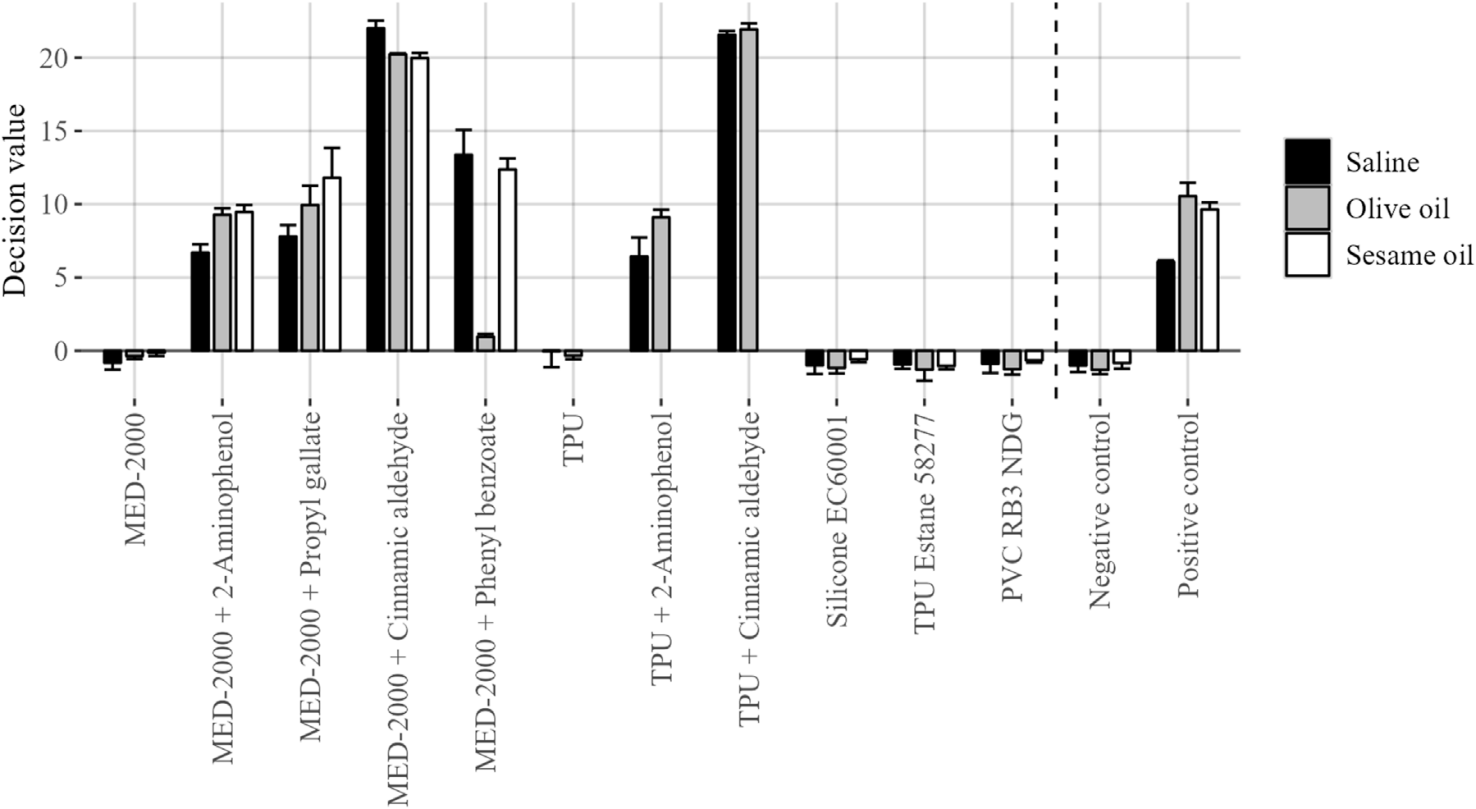
GARDskin classification results of investigated test material extracts. Plotted data corresponds to mean decision values (n = 3), with standard deviation represented by error bars.

Following cell exposures, the conventional GARDskin protocols were followed. Total RNA was isolated from exposed cell cultures and the gene expression levels of the GARDskin GPS were quantified using NanoString instrumentation. Lastly, the GARDskin prediction model was employed to classify each test material extract, with results being summarized in Figure 2. It was concluded that all spiked customized materials were accurately classified as sensitizers, while unspiked customized materials, as well as all commercially available products, were accurately classified as non-sensitizers. Of importance, all classifications of spiked materials as sensitizers match those of the corresponding GARDskin classifications of the pure sensitizing agents, all of which have been assayed previously (2-aminophenol; Forreryd et al., 2016; Forreryd et al., 2023a; Johansson et al., 2017. Propyl gallate; Johansson et al., 2017; Johansson et al., 2019. Phenyl benzoate; Johansson et al., 2017. Cinnamic aldehyde; Johansson et al., 2019). Of further note, all sensitizing agents were accurately classified in all test material extracts, irrespective of the extraction vehicle being used. However, MED-2000 spiked with phenyl benzoate was assigned with DV:s of notably smaller magnitude when extracted with olive oil. A confirmed explanation as to why this is the case is not available, however, it can be hypothesized that the sensitizing agent was more successfully extracted using saline or sesame oil.

Taken together, the adapted GARDskin Medical Device prediction model, which is based on evaluation of both a polar and non-polar extract, would have classified each spiked material as a sensitizer, irrespective of if olive oil or sesame oil were used.

## Discussion

Skin sensitization is, alongside the endpoints of irritation and cytotoxicity, one of the three biological endpoints that must be evaluated for all medical devices (ISO 10993-1, 2018). In the cases of irritation (ISO 10993-23, 2021) and cytotoxicity (ISO 10993-5, 2009), validated *in vitro* methods have been qualified and are well established in routine use. However, skin sensitization testing of medical device products and materials are in practice predominantly being done by the use of *in vivo* experimentation. Nonetheless, the ISO 10993 series of standards explicitly encourages the use of *in vitro* methods, provided such methods are sufficiently validated, as defined by the regulatory context. To this end, ongoing efforts aim to qualify existing and emerging NAM:s for skin sensitization to the applicability domain of medical devices.

In the current study, obtained data demonstrate that GARDskin (OECD, 2023b) can, with few minor adaptations to the conventional protocol, be applied to accurately predict the sensitizing potential of medical device polar and non-polar extracts, prepared according to established extraction procedures described in ISO 10993-12. Furthermore, it was shown that the method is sufficiently sensitive to accurately detect extracted chemicals in such complex mixtures, from two custom materials, spiked with weak, moderate and strong chemical skin sensitizers. Here, custom-made spiked materials were employed in the absence of readily available medical devices with known and well-characterized sensitizing properties. Indeed, all spiked materials, being silicone-based or TPU-based, respectively, were classified as sensitizing hazards by the GARDskin method, based on response-signals originating from both polar and non-polar extracts. Of further note, both sesame oil and olive oil, which are commonly used non-polar extraction vehicles for skin sensitization testing of medical devices, were demonstrated to work with the assay. While all test material extracts produced from spiked materials were individually classified as positive by the GARDskin prediction model, this also shows that the adapted GARDskin Medical Device prediction model classified all spiked materials as positive, in line with the expectation, irrespective of which non-polar vehicle was considered by the prediction model. Lastly, the specificity of the method was further explored by testing commercially available silicone and TPU products, as well as a PVC product, using identical protocols. Here, all commercial materials were classified as non-sensitizers.

To the best of our knowledge, this is the first time an OECD TG 442E method for skin sensitization has been shown to be compatible with ISO 10993 established extraction procedures and vehicles. However, recent work investigates the response in both DPRA (OECD TG 442C) and LuSens (OECD TG 44D) when testing a set of commercially available medical devices (Svobodová et al., 2021). While the specificity from such testing is encouraging, a systematic evaluation of positive response signals from testing of expected positive control materials is lacking. In addition, examples of similar work include the use of non-validated methods and experimental setups (McKim et al., 2012; Coleman et al., 2015; Pellevoisin et al., 2021). The lack of validated *in vitro* methods explored for their applicability for testing of medical devices may, at least in part, be attributed to the difficulties typically associated with applying oil-based test material extracts to submerged cell cultures. Indeed, in work by Svobodova et al., the organic solvent used for extractions were DMSO (LuSens) or excluded altogether (DPRA). In the present study, oil-based extracts were added to the surface of cell culture-wells, without any attempts to further mix the oil-water phases. Nonetheless, it was concluded that positive signals were obtained also from such oil-exposed samples. This may partially be explained by the low limit of detection of GARDskin, which has been demonstrated to be able to detect sensitizing agents at concentrations of only a few ppm (Gradin et al., 2021). In addition, GARDskin has been shown to be widely applicable for hydrophobic substances (Forreryd et al., 2023a), which together with herein presented data may suggest that oil-water phase separation may not necessarily constitute a hurdle. However, it must be considered that these conclusions are, so far, based on a small set of sensitizing agents, all of which are at least in part soluble in water, with LogP-values ranging up to 3.59. Further testing of a wider chemical domain is warranted, in order to explore if these conclusions hold true also for even more hydrophobic substances.

Even though classifications of all materials were successful and concordant across the different solvents, a notable observation was made for MED-2000 spiked with phenyl benzoate, for which the olive oil generated DV:s with significantly lower magnitude, compared to both sesame oil and saline. No apparent explanation as to why is readily available. Indeed, olive oil and sesame oil have a similar composition (Burnett et al., 2017) and therefore also their extraction efficiencies may be assumed to be comparable. Nonetheless, potential hypothesized explanations may include differential extraction efficiencies, unique solvent components with quenching effects, or deviating responsiveness in exposed cell cultures. Repeated testing and chemical characterization of the extracts may be warranted to reach firm conclusions.

It may be noted that the term ‘medical device’ may encompass a wide range of products, with varying physical and physiochemical properties. While the herein presented work is specifically aimed to address issues arising when testing solid materials, a number of medical devices, such as readily available complex mixtures and substance-based devices, are not expected to be advantageously tested using extraction protocols. In such instances, it is important to consider the applicability domain of each test method, to ensure appropriate testing of specific products. To this end, the ability of GARDskin to accurately detect skin sensitizing properties in complex samples, such as mixtures and formulations (Corvaro et al., 2023), UVCBs (Forreryd et al., 2023a) and metals (Forreryd et al., 2023b) have been demonstrated. In addition, applicability has been characterized across a wide chemical domain (OECD, 2023b). As such, it is expected that GARD skin may be widely applied to address the testing needs of a wide range of medical devices with varying properties.

Moving forward, the acceptance of NAM:s for the use of testing of skin sensitizing properties of medical devices will ultimately depend on regulating bodies, which in turn will depend on both the regulatory context and the geographical location of the relevant market. In order to harmonize all efforts to reach acceptance across all markets, experts within the ISO technical committee (TC) 194 recently published a technical specification describing the validating qualification steps foreseen to be required (ISO/TS 11796, 2023). This guidance may be viewed as complementary to guidance provided by the OECD for testing of chemicals (c.f. OECD Guidance Document No. 1 and No. 34). Using the definitions provided in ISO/TS 11796, the herein presented work may be regarded as a ‘feasibility study’, detailing all required amendments to the conventional GARDskin protocols, as well as demonstrating functionality. As such, a ‘pre-validation study’, comprising testing of a set of negative reference material extracts (polar and non-polar) spiked with a known concentration of a chemical skin sensitizer, as well as a ‘validation study’, comprising additional testing of a smaller set of test items in an inter-laboratory exercise, are expected to follow.

In conclusion, we here show how the conventional protocols of the GARDskin method for assessment of chemical skin sensitizers can be adapted to comply with relevant ISO 10993 standards for testing of solid materials. We demonstrate the functionality of the amended protocols by accurate skin sensitizing hazard classifications of custom produced solid materials, spiked with sensitizing agents. Lastly, we confirm the specificity of the proposed method by accurate non-hazard classifications following testing of commercially available solid materials. The herein presented results constitute an important step towards the replacement of *in vivo* testing of medical devices.

## Data Availability

The raw data supporting the conclusion of this article will be made available by the authors, without undue reservation.
